# Supplementary material for: Microscopy Nodes: versatile 3D microscopy visualization with Blender
Source: EMBO Rep. 2026 Jan 5;27(3):581–97. doi: 10.1038/s44319-025-00654-8 (PMC12894756; doi:10.1038/s44319-025-00654-8)
Supplement: Supplementary file 2 — Movie EV1 [file 44319_2025_654_MOESM2_ESM.zip › Movie EV1.docx]

Movie EV1. **Time-lapse render of a mitotic cell.** *Microscopy Nodes renders 5D microscopy stacks.* A mitotic cell with cell (yellow) and chromosome (purple) segmentations is shown, with a volumetric render of the fluorescent signal showing DNA (cyan). The grid is 10 µm.
