# Supplementary material for: Microscopy Nodes: versatile 3D microscopy visualization with Blender
Source: EMBO Rep. 2026 Jan 5;27(3):581–97. doi: 10.1038/s44319-025-00654-8 (PMC12894756; doi:10.1038/s44319-025-00654-8)
Supplement: Supplementary file 3 — Movie EV2 [file 44319_2025_654_MOESM3_ESM.zip › Movie EV2.docx]

Movie EV2. **Video of RPE1 cell expansion microscopy.** *Microscopy Nodes allows for animation of camera and visualization parameters.* The camera zooms in on the centrioles (acetylated tubulin, cyan) while the microtubules (αβ-tubulin, white) fade out. The nucleus, stained with Hoechst, is shown in pink. The acetylated tubulin subsequently fades out to be replaced with a purple model of the acetylated centriole, which is then appended with other centriolar protein elements (like pericentriolar material, subdistal appendages, and linker). The biological grid is 5 µm, the expanded grid is 20 µm.
