# Supplementary material for: Microscopy Nodes: versatile 3D microscopy visualization with Blender
Source: EMBO Rep. 2026 Jan 5;27(3):581–97. doi: 10.1038/s44319-025-00654-8 (PMC12894756; doi:10.1038/s44319-025-00654-8)
Supplement: Supplementary file 4 — Movie EV3 [file 44319_2025_654_MOESM4_ESM.zip › Movie EV3.docx]

Movie EV3. **Video showing U-Ex-STED centrioles with annotation.** *Blender with Microscopy Nodes allows simultaneous presentation of 3D volumetric data and coarse or molecular models.* U*-*Ex-STED microscopy data of the centrioles, where components of three different subcomplexes are stained: the inner γ-tubulin pool in the centriole lumen (red), acetylated tubulin in the MT wall (cyan), and Cep63 in the scaffolding torus (yellow). A model of these subcomplexes, derived from various imaging modalities reported in the literature, gradually appears as an overlay.
