# Supplementary material for: Microscopy Nodes: versatile 3D microscopy visualization with Blender
Source: EMBO Rep. 2026 Jan 5;27(3):581–97. doi: 10.1038/s44319-025-00654-8 (PMC12894756; doi:10.1038/s44319-025-00654-8)
Supplement: Supplementary file 5 — Movie EV4 [file 44319_2025_654_MOESM5_ESM.zip › Movie EV4.docx]

Movie EV4. **Video showing a FIB-SEM dinoflagellate and its segmentations.** *Microscopy Nodes shows the context of volumetric EM by showing electron-sparse regions as transparent.* The render goes through a Z-stack showing separately sliced FIB-SEM volume and trichocyst segmentations (multicolor), to then rotate and show theca segmentation (blue), chloroplast segmentation (green), mitochondria segmentation (yellow).
