# Supplementary material for: Microscopy Nodes: versatile 3D microscopy visualization with Blender
Source: EMBO Rep. 2026 Jan 5;27(3):581–97. doi: 10.1038/s44319-025-00654-8 (PMC12894756; doi:10.1038/s44319-025-00654-8)
Supplement: Supplementary file 6 — Expanded View Figures [file 44319_2025_654_MOESM6_ESM.pdf]

Expanded View Figures

Figure EV1. Shader configuration in Fig. 2.

A repeat of the figure panel of Fig. 2 and accompanying a screenshot of the shader configurations. Data were loaded from https://uk1s3.embassy.ebi.ac.uk/idr/share/microscopynodes/FIBSEM_dino_masks.zarr with emission off, with the data channel as volumetric, and the chromosomes as label mask. All changes made from the default Microscopy Nodes shader settings are outlined in magenta. (**A**) The dense orthographic render of Fig. 2B is made by setting "Alpha Baseline" high (here 10), making the block very dense, and turning "Clip Min" and "Clip Max" both off, showing the maximum and minimum values as dense black and white, instead of transparent. Additionally, we adjusted the pixel intensities to show the nuclear tunnels and inverted the colormap. The inverted colormap can either be achieved by right-clicking the colormap box, or be set as the default in the add-on preferences. (**B**) To generate the sparse volume render, the pixel intensity window that is selected is a window that corresponds to the EM densities of membranes, "Clip Min" and "Clip Max" are ticked, rendering all data outside of the pixel intensity window as transparent. Additionally, this render uses high settings for the scattering of light in the render (Render Properties > Volumes > Max Steps) and how many times a ray of light bounces (Render Properties > Light Paths > Transparent/Total/Volume). This allows the cavities to be clearly darker as light scatters inside it. Here, other settings, such as scattering anisotropy, defining whether light scatters more forward or backward, have been left unchanged. (**C**) The same render as in (**B**), with included masks. These are loaded from a channel of the dataset containing label masks for the chromosomes (bitmap images that contain separate values for each object), and loaded as label mask with emission off and the colormap Tab10 (this can be selected from the loading window, or by right clicking on the colormap here), the only change made is to reduce the opacity to 0.2, to allow us to still see the nuclear tunnels.

© European Molecular Biology Laboratory and Brady Johnston

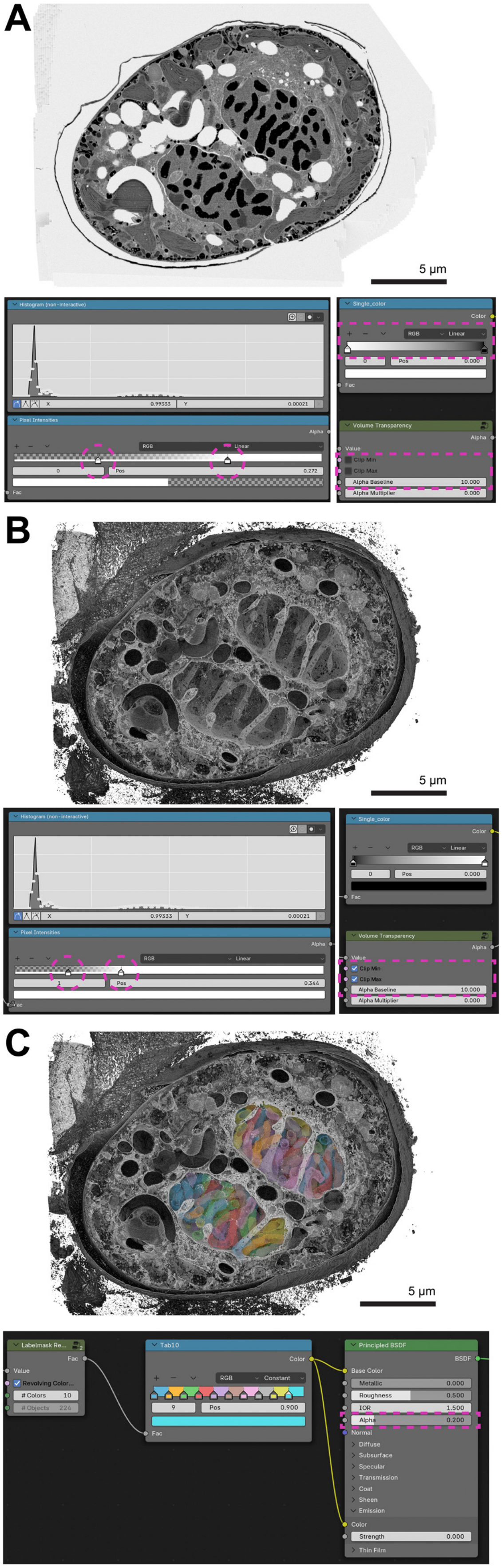

© European Molecular Biology Laboratory and Brady Johnston

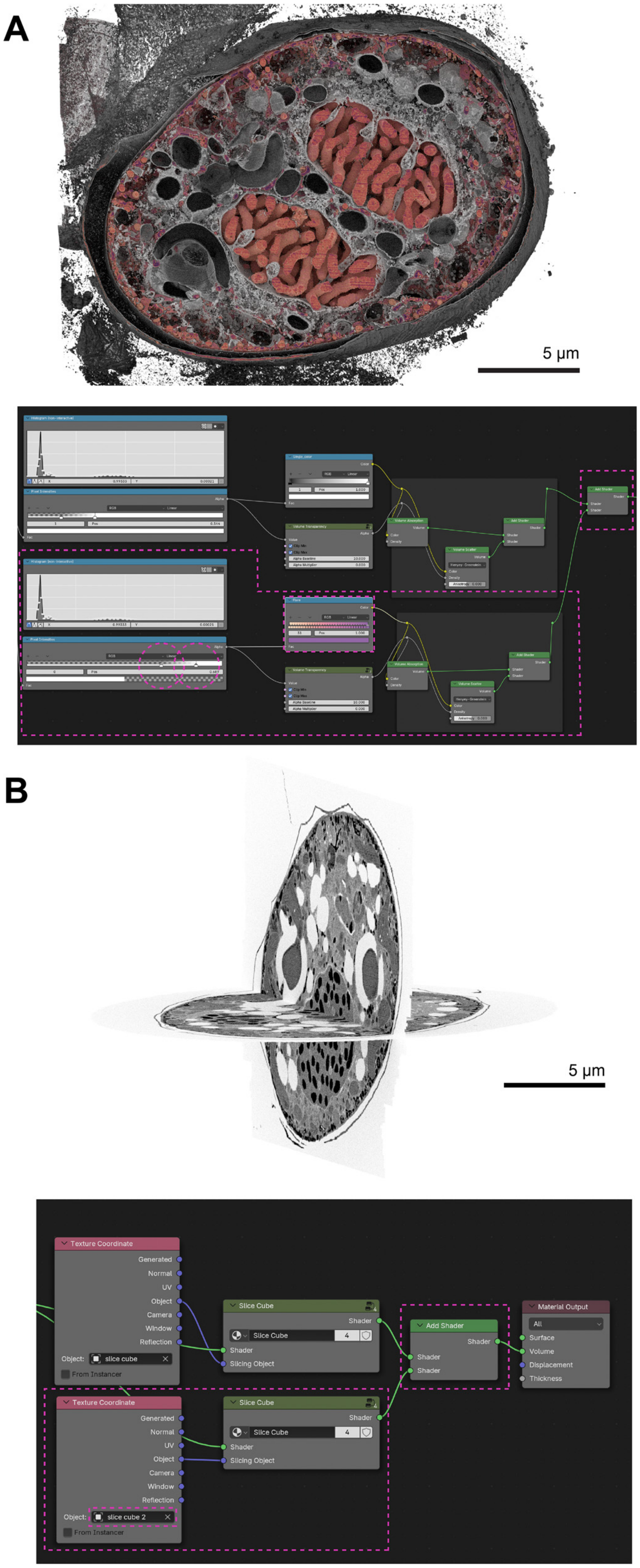

 © European Molecular Biology Laboratory and Brady Johnston

Figure EV2. The configuration of visualization modes is very flexible.

(**A**) Structures with different EM densities can also be displayed with separate colormaps in the same visualization. This is done by duplicating the parsing of data. Here, membranes are visualized in gray (settings as described in Fig. EV1B), and chromosomes are visualized by selecting the appropriate (higher) pixel intensity window and adjusting the colormap (magenta boxes). The colormap for this region is set to seaborn::flare, which can be found under the right click on the lookup table. (**B**) This is a shader setup to show how to add a second slicing cube. This uses the same visualization as used in Figs. 2B and EV1A. Here, the normal slicing cube is made very thin in one axis to make it into a single slicing plane, and copied to make a second slicing cube object. In the shader of the volume, we copied the slicing mechanism and pointed it to the new cube. We combine the two slices with an "Add Shader" node.

© European Molecular Biology Laboratory and Brady Johnston

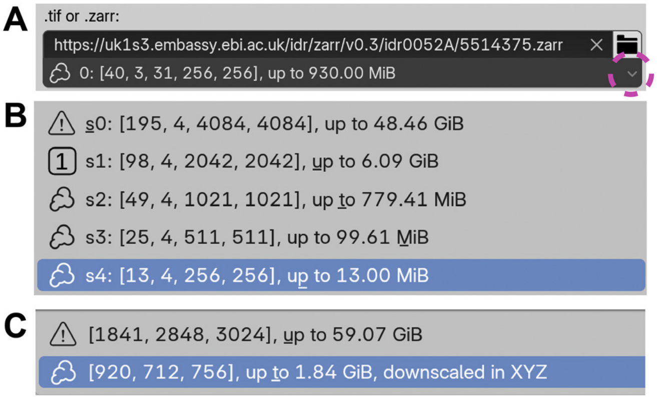

Figure EV3. Selection of data scales allows switching between computational complexities.

(**A**) The location of the scale selection window, directly under the selected path, the button to show all scale options is circled in magenta. (**B**) Selection window for an OME-Zarr with presaved scales. The warning icon is for scales that only work in the raytraced renderer, the 1 icon is if only one channel fits in the rasterized rendering. (**C**) Selection window for Tif scales. Smaller scales than 4 GiB per frame are dynamically generated by downsampling. The algorithm prioritizes downsampling in Z (twofold), then does XY downsampling (fourfold), until the dataset is less than 4 GiB per frame.

© European Molecular Biology Laboratory and Brady Johnston

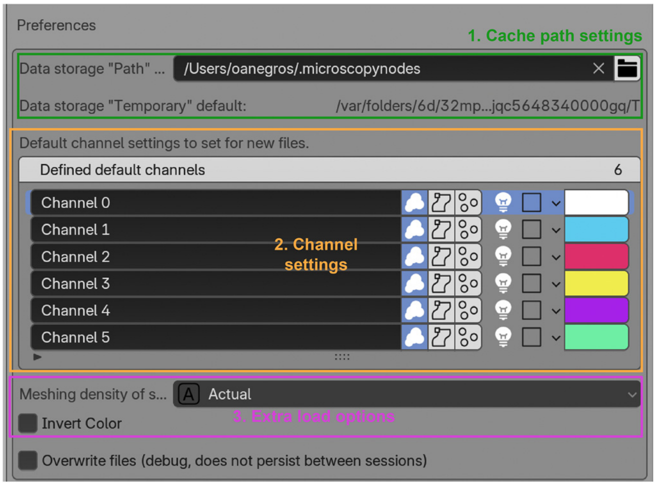

Figure EV4. Default load settings can be configured in preferences.

The preferences can be found under Edit > Preferences > Add-ons > Microscopy Nodes. (1) This allows showing the default static path for local storage, and the auto-generated temporary path used. These are used when the “Data Storage” in the main Microscopy Nodes is set to “Path” and “Temporary”, respectively. (2) The default channel settings are used whenever a new filepath or URL is entered. This allows users to set up their Microscopy Nodes environment to their usual use cases, including colors, or how data is organized. (3) Extra load options comprise the default mesh density of masks and surfaces (default matches the vertex density to the pixel size, but coarser meshes can be selected), and an option to invert color by default.

© European Molecular Biology Laboratory and Brady Johnston
